# IMI—2025 Digest

**DOI:** 10.1167/iovs.66.12.27

**Published:** 2025-09-11

**Authors:** Nina Tahhan, Mark A. Bullimore, Xiangui He, Lisa A. Ostrin, Timothy J. Gawne, Kate L. Gifford, Pauline Kang, Ian Morgan, Aude Couturier, Kyoko Ohno-Matsui, Nicola S. Logan, Ian Flitcroft

**Affiliations:** 1 Brien Holden Vision Institute, Sydney, New South Wales, Australia; 2 School of Optometry and Vision Science, University of New South Wales, Sydney, Australia; 3 University of Houston, College of Optometry, Houston, Texas, United States; 4 Shanghai Eye Diseases Prevention and Treatment Center/Shanghai Eye Hospital, School of Medicine, Tongji University, Shanghai, People's Republic of China; 5 Department of Optometry and Vision Science, University of Alabama at Birmingham (UAB), Birmingham, Alabama, United States; 6 Queensland University of Technology, Brisbane, Australia; 7 Research School of Biology, Australian National University, Canberra, Australian Capital Territory, Canberra, Australia; 8 State Key Laboratory of Ophthalmology, Zhongshan Ophthalmic Center, Sun Yat-Sen University, Guangzhou, China; 9 French Myopia Institute, Ophthalmology Department, Foundation Adolphe de Rothschild Hospital, Paris, France; 10 University Paris Cité, Ophthalmology Department, Assistance Publique – Hôpitaux de Paris, Paris, France; 11 Department of Ophthalmology and Visual Science, Tokyo Medical and Dental University, Tokyo, Japan; 12 School of Optometry, Aston University, Birmingham, United Kingdom; 13 Mater Misericordiae Hospital and Technological University Dublin, Dublin, Ireland

**Keywords:** myopia, binocular vision, pre-myopia, adult myopia, emmetropization

## Abstract

As myopia research expands in scope and complexity, the International Myopia Institute (IMI) continues to provide timely, evidence-based consensus guidance through its biennial white papers and digests. The IMI 2025 Digest delivers targeted updates in areas identified for revision since the previous digest: definitions and classification of myopia, clinical management guidelines, risk factors, accommodation and binocular vision, experimental models, and onset and progression in young adults. A major focus is the evolving concept of pre-myopia, including a refined understanding of hyperopic reserve as a predictive marker, population-specific thresholds, and the distinction between risk and predictive factors. Emerging evidence supports earlier identification and targeted interventions during the pre-myopic phase to delay onset. Clinical management updates highlight that delaying onset by even 1 year may have greater lifetime benefit than multiple years of progression control. Other updates address the limited causal inference of many current risk factor studies, new molecular insights from experimental models, and evidence that a subset of young adults, particularly with high myopia, continue to progress at meaningful rates. Collectively, the IMI 2025 Digest underscores the importance of proactive, individualized myopia care, equipping clinicians with current, evidence-based guidance while supporting continued research and innovation.

As the global body of myopia research continues to grow, both in volume and in complexity, and the range of myopia control interventions expands, the International Myopia Institute (IMI) remains committed to supporting clinicians and researchers by providing timely, accessible, and evidence-based guidance. The IMI achieves this through the publication of peer-reviewed white papers and digests that synthesize the latest developments in the field. These publications are developed through an expert-led consensus process, bringing together internationally recognized leaders in their respective fields to ensure both scientific rigor and practical relevance.

The inaugural IMI white paper series was published in 2019, with subsequent series released biennially. Each white paper addresses a specific and critical area of myopia science and clinical care. Given the ongoing and rapid evolution of knowledge in these domains, the IMI also publishes a biennial Digest, offering concise updates to previously published topics where significant advancements have occurred.

In this 2025 IMI Digest, updates on the following key areas are presented:

• Definitions and classification of myopia
• Clinical management guidelines
• Risk factors for myopia
• Accommodation and binocular vision in myopia development and progression
• Experimental models of emmetropization and myopia
• Onset and progression of myopia in young adults

Whereas previous digests have provided substantial updates on interventions for myopia onset and progression, the rapid expansion of research in this area, and, most importantly, its direct implications for clinical decision making and patient outcomes, has prompted the decision to address this topic in a separate, dedicated white paper rather than summarizing it within the current digest.^1^ This marks the first time a white paper topic has been revisited in a subsequent series, underscoring the growing clinical importance of this field and the need for comprehensive up-to-date guidance for practitioners. Similarly, due to the increasing volume and complexity of genetic research related to myopia, a new genetics white paper^2^ has also been developed as a standalone report to comprehensively cover this evolving field. This marks the first time that topics from prior white papers have been revisited in subsequent series, underscoring their growing importance and the need for comprehensive up-to-date guidance for practitioners.

Not all topics from previous white papers are included in every digest. Rather, the IMI Digest selectively highlights those areas where recent research offers the most substantial and clinically relevant insights. A key focus in this edition is the evolving concept of pre-myopia, now addressed in greater depth within the sections on definitions and classification and clinical management guidelines. Although pre-myopia has been discussed in previous digests, new evidence with direct implications for clinical practice justifies its expanded coverage in this edition. Other areas featured here reflect meaningful advances that are of relevance to researchers and practitioners alike.

## Definitions and Classification of Myopia

This section of the digest provides two key updates: the first relates to the evolving definition and clinical criteria for pre-myopia, and the second addresses updated nomenclature for surgical retina, as agreed upon by experts during the inaugural Myopia Society Meeting held in Paris in June 2024. An additional topic on terminology distinguishing myopia “correction,” “control,” and “management” was originally intended to be included in this section. However, given its conceptual importance and relevance across clinical and research contexts, it was determined that the topic warranted focused attention. As such, it is presented in a separate consensus editorial within this 2025 special issue of IMI white papers.^3^

### Pre-Myopia

#### Hyperopic Reserve

During refractive development in childhood, emmetropization involves a gradual reduction in hyperopia, with some children eventually progressing to myopia.^4^ Recent studies on pre-myopia have increasingly focused on the concept of “hyperopic reserve,” which relates to the evidence that cycloplegic spherical equivalent refractive error is the single best predictor of future myopia onset.^5^^–^^10^ Hyperopic reserve refers to the age-appropriate level of hyperopia that offers a protective buffer against the development of myopia, with higher levels required at younger ages to reduce the risk. Low hyperopic reserve is a central feature of the concept of pre-myopia, as noted in the 2023 Digest,^11^ and has been a key indicator in recent studies.^12^^–^^15^

The current upper refractive error threshold for pre-myopia, set by IMI at +0.75 diopter (D),^16^ was based on data from the Collaborative Longitudinal Evaluation of Ethnicity and Refractive Error (CLEERE) study using a multi-ethnic cohort based in the United States.^9^ Recent studies in Asia have shown variations in hyperopic reserve estimates across different age groups.^5^^–^^8^^,^^17^^–^^19^ However, methodological differences between studies – including differences in study design, population characteristics, statistical methods, and follow-up periods – make direct comparisons difficult. A recent study using comparable methods in China and Europe found that although refractive error is the strongest predictor of myopia onset in both populations, Chinese children appear to need a greater hyperopic reserve than European children to avoid becoming myopic.^18^

#### Risk Factors Versus Predictive Factors

As previously defined by IMI,^16^ pre-myopia is “a refractive state of an eye of ≤ +0.75 D and > −0.50 D in children where a combination of baseline refraction, age, and other quantifiable risk factors provide a sufficient likelihood of the future development of myopia to merit preventative interventions.” This definition highlights that, in addition to evaluating a child's refractive error at any given age, further assessment of their risk of developing myopia should consider the interaction of additional factors, such as parental myopia and the predictive power of these risk indicators. The terms risk factors and predictive factors are sometimes used interchangeably, but they serve different purposes,^20^ especially when translating research into clinical decision making. Risk factors (explored in the IMI—Risk Factors for Myopia paper, published in 2021)^21^ are variables that are associated with an increased likelihood of developing a condition. These are often identified in epidemiological studies and help explain why certain groups have higher rates of myopia than others. For example, reduced time spent outdoors and having two myopic parents are well-established risk factors for myopia.^21^ These factors are important for understanding disease causation and designing population-level public health strategies, for instance, promoting outdoor activity in schools to reduce overall myopia prevalence. However, not all risk factors are useful for predicting who will develop myopia on an individual level.^20^

Unlike risk factors, which are associated with a higher likelihood of developing myopia but do not account for an individual's current status, predictive factors are measurable at a specific point in time and can be used to estimate short-term risk, for example, over 1 or 2 years. For example, a child may have several background risk factors, such as having two myopic parents and spending little time outdoors, but if their current refractive error is strongly hyperopic for their age, their near-term risk of developing myopia may still be low. This is because predictive models incorporate current refractive status, which reflects the cumulative effect of underlying risk factors and environmental influences up to that point.

There have been a number of studies investigating predictive factors for myopia onset in pre-myopic children, and, in recent years, a number of online risk calculators have become available to help clinicians assess a child's risk of developing myopia.^18^^,^^22^^–^^26^ All calculators use refractive error as the main predictor, but the use of other predictive factors varies and clinicians should consider how well the study population characteristics align with the individuals being assessed when considering their choice.

Normative axial length growth charts have been developed for specific ethnic groups and sex and can be used to identify children whose axial lengths fall within a higher percentile range associated with higher prevalence and severity of myopia later in life.^27^^–^^31^ Although axial length measures taken at a given point in time can be a helpful guide, particularly when interpreted against normative charts, its predictive capability in determining whether an individual child will develop myopia has been found to be lower than cycloplegic refractive error at a given age.^5^^–^^10^^,^^18^ A more promising, yet underexplored, predictive marker is the *rate* of axial elongation in an individual child over time. Additional longitudinal data are needed to help clinicians determine elongation rates that exceed typical patterns seen in children who remain emmetropic.

#### Pre-Myopia Intervention Evidence

The final part of the original pre-myopia definition, that is, “to merit preventative interventions,” is often neglected but is now becoming more clinically relevant. Increasing time outdoors is well established as a preventative measure for myopia, with relative reductions in myopia incidence ranging from 16.5% to 50%.^32^^–^^34^ One recent study in Taiwan found that a group of pre-myopic children aged 7 to 11 years who were encouraged to go outdoors during recess had a lower incidence of myopia than a control group (19.6% vs. 37.8%). However, the risk of myopia onset was higher in pre-myopic children than those who were not pre-myopic^35^ (as might be expected due to the relatively higher hyperopic reserve in children who were not pre-myopic).

New evidence supports the benefits of more direct interventions, such as low-dose atropine, repeated low-level red-light (RLRL), and novel spectacle designs for delaying myopia onset in high-risk children. These are discussed in further detail in the IMI interventions paper in this 2025 series.^1^

### Nomenclature for Surgical Retina

The inaugural Myopia Society Meeting held in Paris in June 2024 brought together international myopia and retinal imaging specialists to enhance global collaboration, present new research, and refine clinical guidelines and definitions for surgical retinal conditions.

A central achievement of this gathering was the establishment of a standardized nomenclature for myopic traction maculopathy (MTM). The international expert panel selected by the Myopia Society systematically reviewed existing MTM classifications and their limitations. By incorporating recent optical coherence tomography (OCT) findings, they developed a clinically relevant hierarchical nomenclature aimed at accurately reflecting lesion severity, predicting visual outcomes, and guiding clinical management.

The expert panel recognized that MTM arises and progresses primarily due to several inter-related biomechanical forces: posterior staphyloma-induced outward scleral expansion, vitreomacular traction commonly associated with perifoveal posterior vitreous detachment, residual cortical vitreous remnants, epiretinal membrane contraction, intrinsic limitations in the elasticity of the inner limiting membrane, reduced axial stretch capability of retinal vessels compared to adjacent retinal tissue, and paravascular vitreous adhesions. Whereas the precise magnitude and directional vectors of these forces remain challenging to quantify clinically, understanding their roles helps inform management strategies and prognosis.

The newly agreed-upon classification identifies four primary MTM manifestations, prioritized based on visual impact: (1) myopic macular detachment, (2) full-thickness macular hole, (3) lamellar macular hole, and (4) retinoschisis. The classification uses the term “myopic macular” before the most severe lesion, followed by other coexisting lesions in descending order of severity—for instance, “myopic macular detachment with full-thickness hole and retinoschisis.” Additionally, the expert panel established standardized OCT imaging protocols to ensure consistency in diagnosis and monitoring disease progression (the full report is yet to be published).

This OCT-based anatomic nomenclature aimed to address previous classification shortcomings by emphasizing clinical relevance and visual prognosis, thereby standardizing lesion descriptions and enhancing clinical utility. By providing a clear framework for describing complex MTM presentations, this nomenclature facilitates disease monitoring, improves clinical decision making, and enables consistent research reporting.

## Clinical Management Guidelines

The new white paper, *IMI-Interventions for Controlling Myopia Onset and Progression 2025*,^1^ builds on the Clinical Management Guidelines published in 2019, and the two Digest updates in 2021 and 2023, to provide an overview of the latest knowledge on myopia treatment and management strategies to support evidence-based practice. With the ongoing advancements in myopia science, treatment options, and management paradigms, this Digest edition explores the following key areas: preventative management; proactive myopia management including guidance on who to treat, when to initiate treatment and how to manage treatment; new approaches in understanding and describing myopia control efficacy; and long-term myopia management, including the vital importance of ocular health monitoring.

### Preventative Myopia Management

Delaying the onset of myopia can significantly reduce the final degree of myopia in adulthood. A recent analysis examining the impact of age at myopia onset found that each year of delayed onset was associated with 0.68 D to 0.97 D less myopia in the final recorded refractive error. A 1-year delay in myopia onset, for a child of East Asian ethnicity, can lower the final level of myopia by the equivalent of 2 to 3 years of treatment with current myopia control modalities. The effect was meaningful but less pronounced for non-East Asians.^36^

When prescribing any treatment for pre-myopia (see earlier pre-myopia section and 2025 IMI white paper on interventions),^1^ clinicians must balance the potential risks and costs of treatment with the anticipated benefits of treatment. Although preventing myopia and its associated risk of irreversible vision loss^37^ offers clear benefits, there is no certainty that a pre-myopic child will develop myopia or that early intervention will prevent its onset. Therefore, careful evaluation of individual risk levels along with other patient factors such as motivation, lifestyle and economic factors, are important in the overall decision making.

### Proactive Myopia Management: Who, When, and How to Treat

Since the publication of the first IMI Clinical Management Guidelines in 2019,^38^ the evidence base for myopia treatments and their impacts have expanded significantly, further strengthening the motivation to slow the rate of myopia progression and axial elongation in order to reduce the associated risks of sight-threatening ocular diseases.^36^^,^^38^^,^^39^ In addition, those with higher levels of myopia report poorer quality of life than those with lower levels of myopia.^40^ This includes greater limitations in daily activities, increased visual dependency on corrective lenses, heightened concern about long-term eye health risks such as retinal detachment or myopic maculopathy, and greater emotional and functional burden related to their vision.^40^ Recognition of myopia management as standard of care has also grown, with a resolution by the World Council of Optometry in 2021^41^ and a World Society of Paediatric Ophthalmology and Strabismus Consensus Statement in 2023.^42^ These international organizations concur that proactive myopia management with evidence-based treatments, rather than single vision correction, is a clinical imperative for short-term functional and long-term ocular health benefits.

Recent long-term data demonstrate the sustained efficacy and good acceptance of several myopia control interventions. Dual-focus soft contact lenses,^43^ and defocus incorporated multiple segment (DIMS) spectacle lenses^44^ have been evaluated over 6 years, including participants up to 18 to 19 years of age, whereas highly aspherical lenslet (HAL) spectacle lenses have shown positive outcomes over 5 years.^45^ These studies are no longer randomized controlled trials (RCTs), as the original control groups were switched to the intervention after 2 or 3 years. However, they continue to show an accumulation of treatment effect^43^ and no evidence of rebound following treatment cessation.^44^ Six-year data on dual-focus daily disposable soft contact lens wear has also shown no measurable impact on ocular health compared with baseline non-lens-wearing measures, assessed by biomicroscopy examination of the external adnexa and ocular surface, indicating a high long-term safety profile.^46^

It is beyond the scope of this paper to review treatments (refer instead to the IMI - Interventions for Controlling Myopia Onset and Progression 2025),^1^ but it is important to note that, with increasing availability of current treatments and evidence on emerging treatments, the clinician has more options to personalize treatment to suit the child's individual optical and ocular characteristics; lifestyle, sports and hobbies; and personal and family capacity.^47^ Because optical correction is needed by the myopic child, it is reasonable to consider evidence-based optical interventions as first-line treatment for the dual benefit of myopic refractive error correction and control of myopia progression, where suitable and available. This approach is supported by current knowledge that numerous treatments demonstrate comparable efficacy in slowing myopia progression.^47^ Furthermore, subjective wearer experience has been shown to correlate with greater wearing time^48^^–^^51^ and increased wearing time is associated with improved myopia control efficacy.^52^^–^^54^ Hence, it is essential to prioritize patient-centered factors in clinical decision making to optimize treatment compliance and achieve the best myopia control outcomes.

The initial IMI Clinical Management Guidelines Report^38^ highlighted the importance of informed and shared decision making in myopia management. This is especially critical when treatments are used off-label. Families should be supported to make educated decisions through clear, balanced communication about the benefits, risks, and limitations of available options, with age-appropriate involvement of the child or young person.

### Understanding Treatment Efficacy: New Approaches

There are various approaches that have been used in the literature to present myopia control efficacy (see the 2025 IMI white paper on interventions).^45^ One approach to evaluating treatment efficacy considers the proportion of axial elongation attributable to “normal” emmetropic growth, often referred to as “physiological” or “compensated” growth.^55^^,^^56^ For example, a recent study evaluating dual-focus soft contact lenses used emmetropic growth as a reference to differentiate physiological^57^ from abnormal myopic elongation. Children wearing single vision contact lenses showed axial elongation similar to that of untreated myopes, whereas those in dual-focus lenses exhibited eye growth more consistent with emmetropic norms. A recent analysis of the HAL spectacle lens technology similarly concluded that in children wearing these spectacles full-time (at least 12 hours per day, 7 days per week), the eye growth pattern of approximately 90% of the children was similar to or slower than that of non-myopic children, compared with only about 10% of children wearing single vision lenses full time.^58^

While referencing emmetropic eye growth provides valuable context for understanding treatment effects, RCTs remain the gold standard for evaluating myopia control treatment efficacy. Given the ethical and methodological challenges of using single vision lenses as controls, future studies should ideally compare new interventions against established myopia control treatments.

### Long-Term Myopia Management

The clinical picture of long-term success in myopia management is defined by slowed refractive and axial length progression compared to progression without myopia control intervention.^59^ It can also be defined by a treatment that is well accepted by the patient, enabling good compliance, has good quality of vision, has a positive impact on quality of life, and demonstrates sustained efficacy and safety.^60^

Regular assessment of the impact of treatment through cycloplegic refractive error and, where available, axial length measurements, helps evaluate myopia control efficacy.^38^ Although ocular biometers may not be readily available to all clinicians, as axial length and refractive error are highly correlated, it should not deter them from engaging in myopia management.^47^ With the risk of ocular morbidity and visual impairment being highly related to axial length, patients should be informed of the importance of monitoring myopia progression as well as monitoring ocular health to detect any early signs of related ocular disease.

A hospital-based cross-sectional study in Vietnam examined patients aged 12 to 47 years (mean age = 20.4 ± 7.2 years) with high myopia (>6.00 D). Peripheral retinal lesions were present in 43.5% of the participants, central retinal changes in 66%, and peripapillary atrophy in 70%. A greater risk of myopic retinopathy was associated with myopia over 8.00 D, axial length ≥26.5 mm, and age over 19 years.^61^ Another study followed a cohort of children in Singapore for up to 19 years and found that in patients with high myopia (−7.01 ± 1.56 D and 26.53 ± 1.00 mm) who were now aged 21 to 27 years, a tessellated fundus was predicted by earlier age of myopia onset, more myopic refractive error, and longer axial length.^62^ In a separate longitudinal study from Singapore, children with high myopia (mean = −7.01 ± 1.56 D; axial length = 26.53 ± 1.00 mm) were followed for up to 19 years. By early adulthood (aged 21–27 years), the presence of a tessellated fundus was associated with earlier onset of myopia, more severe refractive error, and longer axial length.^62^

According to the international Meta-analysis of Pathologic Myopia (META-PM) classification, tessellated fundus represents grade 1 of myopic macular degeneration (MMD), and is associated with the risk of developing more advanced stages of MMD that are associated with vision loss.^63^ In adults aged 40 to 80 years with any level of myopia who developed myopic macular degeneration, 83% had a tessellated fundus at baseline.^64^ These findings highlight the importance of proactive retinal health monitoring as a core component of long-term myopia management, including in younger patients.

## Risk Factors for Myopia

Although numerous association studies on myopia risk factors have been published since the IMI white paper on this topic,^21^ many still exhibit the methodological limitations previously identified. One common issue is the omission of cycloplegic refraction, which remains the gold standard for accurately assessing refractive error in children, adolescents, and young adults.^65^ Another challenge lies in study design, particularly the use of samples that span wide age ranges. As key exposures such as educational demands and outdoor time vary with age, the most powerful evidence comes from large samples of children of the same age or grade, where confounding by factors that vary with age or grade is minimized.

Methodological limitations are particularly common in the large amounts of data coming from school screenings in China, where cycloplegia is not routinely used. Instead, non-cycloplegic refractive error, supplemented by an additional visual acuity criterion, is now the national standard,^66^ but this is still problematic as a measure of myopia, particularly with younger children up to the age of 7 or 8 years, who often fail the standard visual acuity tests for cognitive rather than visual reasons. In fact, when the appropriate HOTV chart is used, many of the children failing the visual acuity test, show adult level HOTV chart scores.^67^^–^^69^ Another approach that is becoming more common is to apply a more stringent threshold for myopia (generally −0.75 D) when cycloplegia is not used, as suggested in the IMI White Paper on Definitions of Myopia.^16^ Both of these approaches aim to improve the accuracy of referrals based on non-cycloplegic measurements but they require further validation when used to analyze risk factors, particularly regarding whether stricter thresholds may exclude individuals who are genuinely myopic based on the standard ≤−0.50 D definitions. Nevertheless, these methods can be valuable for surveillance and the analysis of secular trends, provided that a consistent methodology is applied.

Data from China has, however, been crucial in resolving one issue. A number of studies, using both cycloplegic and non-cycloplegic measures, have shown that school grade rather than the age of the child correlates best with refractive error.^70^^,^^71^ Strict enrollment rules mean that children who are only 1 day apart in age can be separated by one grade in schooling, whereas, within a grade, ages range over 1 year. Despite this variation in age, younger and older children within a grade are, on average, very similar in refractive error. In contrast, children of a very similar age can differ by 1 year in duration of schooling and differ markedly in refractive error by an amount that effectively defines the average impact of 1 year of education at that grade level in that school system. This should enable future research to more precisely link exposures over 1 school year to changes in refractive error and to more precisely document the impact of interventions to prevent and control myopia at a given level of schooling. An important implication of these findings is that most of the myopic shifts in refractive error after the age of 6 years in children who are not myopic are due to environmental exposures, rather than a normal part of aging. This is consistent with a large body of epidemiological evidence that shows low prevalence of myopia persists where formal education systems are poorly developed.^72^

There have, however, been some important developments in methodology. One of the most important issues in research on risk factors is to identify causal relationships, because these can provide the basis for preventive interventions. The *JAMA* journals have generally insisted on tight restrictions on causal language, limiting its use to results from RCTs and rejecting its use in relation to observational studies.^73^ One of the unfortunate consequences of this position has been that many papers on associations do not consider the potential for a causal link in their findings but terminate any possible discussion by simply stating that association does not prove causation.

Because it is becoming increasingly clear that RCTs on many issues are ethically impossible to perform, the *JAMA* journals have recently initiated a discussion of when causal language can be used in relation to observational studies.^73^ Randomized interventions remain the gold standard in this area, because the randomization process means that potentially confounding exposures will be randomly distributed to the intervention and control arms. However, this sort of evidence is increasingly being supplemented by studies based on Mendelian Randomization (MR).^74^ MR relies on identifying well-established genetic variants –called single nucleotide polymorphisms (SNPs) – that are associated with a particular exposure. Because these genetic variants are randomly inherited, they can help simulate the conditions of a randomized trial, assuming other risk factors are distributed independently of the SNPs in the population. This approach depends on finding well-validated SNPs that are associated with the exposure and the assumption that other relevant risk factors are randomly distributed in the population relative to the SNPs. Examples from myopia research include the use of SNPs associated with increased years of education and SNPs associated with increased myopia to show that increased years of education lead to more myopia, whereas more myopia does not lead to more years of education.^75^ Similarly, whereas observational epidemiology has suggested that there is quite a consistent correlation between more myopia and lower vitamin D levels; SNPs associated with higher vitamin D levels do not lead to less myopia, suggesting that less myopia and higher vitamin D levels are both caused by more time outdoors but are not causally linked.^76^

The technique of Regression Discontinuity Analysis (RDA),^77^ which is widely used in economics and social sciences to study the link between policy changes and outcomes, is now being applied to causal analysis in epidemiology. In this case, there is no randomization, but evidence supporting causality comes from the tight sequential temporal relationship between the policy change and the intervention. This analysis has been used to show that the raising of the school leaving age from 15 to 16 years in England and Wales in 1972 was associated with a significant myopic shift in refractive error, adding support to the hypothesis that increased education causes more myopia. It has also been used to support a causal relationship between the life of school children and sequential increases in myopia as children advance through school grades.^70^^,^^78^^,^^79^

It is important to note that these approaches both have limitations and are frequently based on assumptions that are difficult, if not impossible, to prove. These techniques are most effective when supported by a substantial body of consistent epidemiological evidence, as is the case in myopia research.^80^ When RCTs are not feasible, they can still provide important evidence to support or refute causal hypotheses.

An important additional consideration when assessing causality is whether there is evidence of a plausible pathway linking the exposure to the outcome.^73^ This is the case for time outdoors, where it was initially hypothesized^81^ that this effect was mediated by increased exposure to bright light outdoors, which increased the release of dopamine from the retina,^82^ which then inhibited axial elongation,^83^^,^^84^ based on the existing evidence supporting each step in this hypothetical causal chain. Subsequent studies on the suppression of experimental myopia in animals by bright light gave results generally consistent with the hypothesis.^85^^–^^88^ More recently, it has been suggested that the higher spatial frequency of outdoor scenes may also play a role in the prevention of myopia.^89^

Another recent methodological development concerns the use of mediation analysis to probe whether one risk factor mediates the effect of another. An obvious example would be to ask if time outdoors might mediate the impact of access to green space^90^^–^^92^ on the prevalence of myopia, and mediation suggests that it does. Similarly, the role of decreased time outdoors in the increased myopia seen with more years of education has been examined, with results suggesting that approximately half of the impact of increased education, at least in Europe, may be due to children spending less time outdoors.^74^^,^^93^^–^^95^ As with MR and RDA, mediation analysis is based on assumptions, and investigators need to be aware of the strengths and weaknesses of the approach when carrying out the analysis.

Analysis using instrumental variables is now also being applied to the etiology of myopia.^96^ An instrumental variable is defined as one that influences exposure to a risk factor but does not have a direct effect on the outcome. One of the assumptions is that the instrumental variable does not affect confounding variables. It is important to note that MR is in fact a form of instrumental variable using a genetic parameter. In their paper,^96^ Guggenheim and colleagues used dog ownership as an instrumental variable for time outdoors, on the premise that dog owners generally walk their dog once or twice a day, often accompanied by their children. In their analysis, they confirmed that the children of dog owners reported spending more time outdoors than those who did not, but the effect was only an additional 3 minutes a day.^75^ These children were correspondingly less myopic on average, allowing the authors to estimate that an additional hour of time outdoors per day over the 8 years of the study would be associated with a refractive error that was +0.53 to +0.94 D more hyperopic, whereas an additional hour of reading per day would be associated with a refractive error that was −0.44 to −0.88 D more myopic. In the same analysis, sleep duration was not significantly associated with refractive error. These estimated shifts were substantially larger than those derived from standard regression analysis. This is also the case when using SNPs as instrumental variables in MR analysis,^75^ and with RDA. To put these estimates into context, the intergenerational shift in refractive error observed in China between parents and their more myopic children as young adults was −1.69 D, which could be accounted for by similar shifts due to increased reading time and decreased time outdoors, whereas the children on average undergo a myopic shift of nearly 4 D from the age of 6 years to the end of schooling. The original paper should be consulted for more details of this interesting approach,^96^ which suggests that earlier estimates of effect size may underestimate the true effects of exposures.

Another important technical consideration is how results are reported. It is common to report whether associations are statistically significant, but much less common to report whether the effect sizes are significant and likely to be useful in controlling the prevalence of myopia. As an example, Zhang et al. reported a significant association between population density and the prevalence of myopia, but a plot of the prevalence against population density shows that even at the lowest population densities examined, the prevalence of myopia was high.^97^ This suggests that a common driver for myopia across locations is intensive education combined with limited time outdoors, whereas population density likely plays only a minor modulatory role.

Overall, the causal role of educational exposures in myopia development is increasingly accepted, supported by a large body of observational epidemiology as well as MR and RDA. However, the precise underlying mechanism, whether related to education or near work, remains unclear. Similarly, the protective effect of increased time outdoors is now generally recognized, supported by extensive observational evidence and RCTs.^32^^,^^98^ In particular, large-scale implementation of outdoor time programs within the school curriculum has demonstrated practical effectiveness in reducing myopia onset and progression. In Taiwan, for example, policies promoting outdoor activities during school hours have been associated with stabilization or reversal of myopia prevalence trends in children, as shown in serial population-based surveys and school-based studies.^99^^–^^101^ These programs typically include structured outdoor recess and public health campaigns to encourage children's daily outdoor exposure, contributing to measurable improvements in visual acuity and reductions in myopia incidence.

The potential roles of screen use and sleep patterns in myopia development remain under investigation (discussed also the IMI companion paper on the role of light in refractive development and myopia).^102^ Screen use, ranging from television to smartphones, has been associated with myopia in some studies, but the findings are inconsistent.^103^^,^^104^ It remains unclear whether screen time poses a greater risk than other forms of near work, such as reading. Importantly, the rise in myopia in East Asia predates widespread access to digital devices,^21^^,^^72^ suggesting that reducing screen time alone is unlikely to impact prevalence unless paired with strategies to increase outdoor activity.

Although smartphones became widely available around 2008 to 2009, children with lifelong exposure are still only in their mid-teens, and most were not exposed from infancy. As such, there is limited long-term evidence linking early screen exposure to increased myopia prevalence. Continued monitoring is warranted, particularly in regions with lower baseline prevalence where potential effects may be more pronounced.

The second factor under discussion is a possible causal link between reduced sleep and myopia. Sleep is a complex parameter, and studies to date have examined associations between myopia and short or insufficient sleep duration, late bedtimes, delayed wake times, and sleep disturbances. However, findings to date are inconsistent, as highlighted in several systematic reviews and meta-analyses.^105^^–^^113^ Some MR studies and mediation analysis using instrumental variable approaches have not found strong evidence supporting a causal role for sleep.^96^^,^^114^^–^^117^ One study suggested that chronotype may not be a significant factor.^118^ However, more evidence is needed to rule out potential contributions of sleep.

The current evidence base is limited by several factors, including the reliance on non-cycloplegic refraction, broad age ranges within study populations, and the complex interplay among sleep, time spent outdoors, physical activity, and academic demands. Future longitudinal studies that incorporate cycloplegic refraction in large, age- or grade-specific cohorts, use wearable devices to objectively measure sleep, and ideally include intervention components, would be valuable in better understanding and clarifying these associations.

One important piece of evidence that illustrated the importance of environmental exposures in the etiology of myopia was the surge in myopia that occurred during the coronavirus disease 2019 (COVID-19)^102^ pandemic.^79^^,^^119^^–^^126^ Whereas many of the studies have methodological limitations due to the opportunistic nature of data collection, several reported significant increases in the prevalence of myopia, particularly in younger children following pandemic lockdown measures, although some evidence indicates broader and more lasting behavioral changes in certain regions.^127^ Notably, the Yilan study in Taiwan reported no increase in myopia prevalence among preschoolers participating in a school-based program that involved increased time outdoors.^101^^,^^128^

## Accommodation and Binocular Vision in Myopia Development and Progression

The role of accommodation and binocular vision in driving myopia development and progression remains uncertain. Evidence still points to association rather than causation.^129^ Various interventions aimed at slowing myopia progression in children, such as low-dose atropine and specially designed spectacle and contact lenses that provide relative plus power in the periphery, have the potential to influence accommodative and binocular function. However, whether these alterations also impact myopia progression remains an area of interest.

Multifocal soft contact lenses (MFCLs) create simultaneous distance and near power within the pupil zone, potentially reducing accommodative demand and inducing an exophoric shift at near. Short-term studies in young adults^130^ indicate that dual-focus soft contact lenses increase the lag of accommodation and decrease accommodative facility compared with single-vision soft contact lenses. However, these measurements were taken after just 15 minutes of wear, without assessing long-term adaptation. Another study, also on young adults, reported that both concentric ring and aspheric designs reduced accommodative function and altered vergence, causing an exophoric shift.^131^ Measurements in this study were conducted between 10 and 40 minutes after lens insertion, again without evaluating long-term effects. Because these lenses are typically worn by children over extended periods, it is unclear how these short-term findings translate to clinical practice.

A 2-year prospective study followed 36 children aged 8 to 14 years to evaluate changes in accommodation and binocular function when switching from single-vision spectacle lenses to orthokeratology.^132^ Significant changes in accommodative and binocular function were observed after the switch to orthokeratology, but most parameters stabilized within the first 6 months. Crucially, no association was found between changes in these functions and myopia progression.

The BLINK study found that after nearly 5 years, long-term use of MFCLs in children did not reduce accommodative facility, with no difference observed between MFCL and single-vision wearers when measured with single-vision correction.^133^ Similarly, a 2-year study investigating multi-segment spectacle lenses revealed no changes in phoria status.^134^ Although some changes in accommodation were noted during the 2 years of DIMS lens wear, similar changes were seen in those wearing single-vision lenses.

Atropine is well known to affect accommodative function due to its dose-dependent cycloplegic effects. A study comparing accommodative and vergence function in children using orthokeratology combined with low-dose atropine (0.01%), either intervention alone, or a placebo, found that accommodative measurements, including positive relative accommodation (PRA), binocular accommodative facility, and accommodative lag, improved similarly in both the combination and orthokeratology groups over a 3-month period.^135^ These measures were obtained using subjective methods, except accommodative lag, which was assessed objectively using the monocular estimation method (MEM). In contrast, 0.01% atropine treatment showed no significant effects on accommodative or vergence measurements.

Current data from long-term studies in children consistently indicate that myopia control interventions cause minimal changes to accommodative and binocular functions and that these functions show no correlation with the rate of myopia progression.

## Experimental Models of Emmetropization and Myopia

The period of 2023 to 2024 has seen an enormous amount of research published on myopia using animal models, including mice, guinea pigs, rhesus monkeys, marmosets, chicks, tree shrews, and zebrafish. Myopia work using mice is increasing, especially given more refined techniques and the availability of powerful genetic models. Additionally, a new model of myopia using tree shrews and limited bandwidth ambient light has been developed, and a zebrafish model involving dark rearing has been introduced. Here, the key findings from animal models of myopia published over the previous 2 years are summarized.

### Spectral Composition of Light

As research continues, it has become apparent that the role of light in emmetropization is vastly more complex than was first believed (refer to the IMI companion paper for more details on the role of light in refractive development and myopia).^102^ Animal models provide an opportunity to precisely control environmental light and to genetically manipulate associated pathways.

Animal models have long confirmed that the emmetropizing eye can distinguish between myopic and hyperopic blur.^136^ However, the specific aspects of the retinal image that allow this distinction remain unclear. Many potential visual cues have been proposed, including spherical aberration^137^ and peripheral astigmatism.^138^ Although the system likely combines multiple cues, it is apparent that chromatic cues are critically important.^139^ Vertebrate eyes show significant longitudinal chromatic aberration (LCA), whereby shorter wavelengths focus closer to the front of the eye than longer wavelengths,^140^ which may provide a cue for emmetropization. Additionally, wavelength specific opsins, including novel opsins (discussed below), may play a role in eye growth regulation, as well as relative contributions from traditional rod and cone photoreceptors.

Recent studies have further explored the effects of narrowband ambient light, or spectra that are otherwise significantly altered, on emmetropization. Although there is considerable variability between species and lighting protocols, in general, long wavelengths induce myopia in chicks, mice, fish, and guinea pigs, and the opposite occurs in tree shrews and nonhuman primates (for a review, see Ref. 141). A recent study found that both cyan and red light inhibit axial elongation in zebrafish.^142^ A study in guinea pigs showed that different ambient light spectra had significantly different effects on both axial elongation and scleral metalloproteinase activity.^143^ Periods of blue exposure inhibited the development of experimental myopia in guinea pigs.^144^ Additionally, narrowband blue light inhibited the induction of myopia in chicks,^145^ although, in another study, the effect of narrowband blue light was more complex and could even enhance the development of myopia.^146^ Two recent studies using tree shrews^147^^,^^148^ have confirmed the anti-myopic effect of red light in this species. It remains unclear why red light is effective in slowing axial elongation in tree shrews and nonhuman primates, as well as potentially in humans,^149^^,^^150^ whereas the results from the other commonly used animal models are typically contradictory or mixed.

Using narrowband ambient light is a powerful and technically easy way to explore the effect of chromatic cues on refractive development. However, other aspects of light may also be important; for example, flicker or spatial frequency as a function of wavelength. Imposing a temporal modulation on the ambient light, that is, adding “flicker,” has been shown to affect refractive development. One recent study showed that bright light modulated at a low temporal frequency (0.05 hertz [Hz]) appeared to effectively inhibit lens-induced myopia in guinea pigs.^151^ Another recent study suggested that 2 Hz flickering light could also inhibit induced myopia in mice.^152^ It is not clear why flickering ambient light should affect refractive development, especially as it is superimposed on the substantial local retinal flicker caused by normal eye and head movements. There is at present no widely accepted theory behind these results.

### Spatial Frequency

Spatial frequency characteristics have also been shown to influence eye growth. When placing tree shrews in a small cage with a video display at one end, refractive development can be strongly influenced by altering the spatial frequency distribution as a function of wavelength on the video display, without altering the overall spectrum of ambient light.^153^ A more recent paper in tree shrews further explored the parameters of this effect, finding that a spatial frequency of approximately 1 cycle per degree was most critical in eye growth regulation.^154^ More generally, altering chromatic visual cues without changing the overall spectrum of ambient light could allow more specific investigations of the visual cues guiding refractive development.

### Novel Opsins

Evaluating image focus requires inputs from the classical cone and rod photoreceptors. However, refractive development could also use non-image forming mechanisms. For example, pathways involved in detecting the absolute intensity of the ambient illumination, spectrum, timing across the day, and interaction with circadian rhythms could also be critically important. These processes are mediated by a distinct class of light-sensitive proteins known as opsins, which are found in non-classical photoreceptor cells, including intrinsically photosensitive retinal ganglion cells, amacrine cells, and the retinal pigment epithelium. Although classical visual opsins within rods and cones are responsible for image formation, noncanonical opsins respond to ambient light independently of image formation. To date, three noncanonical opsins have been implicated in refractive development: OPN3 (encephalopsin), OPN4 (melanopsin), and OPN5 (neuropsin).^155^ These opsins initiate light-driven signaling pathways involved in physiological processes, such as circadian regulation, and may play a critical role in modulating refractive development.

OPN4, which is expressed in the intrinsically photosensitive retinal ganglion cells, has been implicated in playing a role in refractive development for some time, likely because of its apparent role in the regulation of retinal dopamine.^156^^–^^158^ In the recent period, further evidence for the involvement of OPN4 in refractive development has been found in rabbits^159^ and guinea pigs.^160^

Roles for OPN3 and OPN5 in refractive development have also emerged, all using the mouse model. OPN5 mediates violet-light early growth response 1 (EGR1) expression in the mouse retina.^161^ OPN3 appears to be required for normal refractive development and GO/GROW response to induced myopia.^162^ Additionally, violet light (360–400 nm) transmission has been shown to have suppressive effects on experimental myopia progression,^163^ suggesting an involvement of OPN5.

From experiments in mice, it has been suggested that violet light may be a viable anti-myopia therapy in humans.^164^^,^^165^ However, attempts at using violet light to slow myopia progression in humans have not been overall significant.^166^^,^^167^ Furthermore, in diurnal species, such as humans and nonhuman primates, violet light is highly filtered (although not completely) by the crystalline lens.^168^ Although the mouse offers powerful genetic tools, it is suggested that further experiments, both in mice and in other species, especially nonhuman primates and other diurnal species with high levels of UV absorbance by the ocular media, are warranted. Refer to the IMI companion paper on the role of light in refractive development and myopia for further details on opsins.^102^

### The Choroid

It is accepted that in emmetropization, the neural retina evaluates focus, as well as potentially spectral characteristics, and a signaling cascade is ultimately transmitted to the sclera to alter fibroblast activity. Thus, the choroid must at least serve as a relay station between the retina and sclera. Alternatively, the choroid might play a more fundamental role. The use of short-term (in the order of tens of minutes) changes in thickness of the choroid is being used as a proxy for longer-term refractive development (for a review, see Ref. 169).

A recent study in mice demonstrated that an alpha-1 adrenergic blocker increased choroidal blood flow and suppressed myopia progression.^170^ Another study in mice also suggested that improved choroidal blood circulation, in this case induced by ginkgo biloba extracts, could suppress myopia.^171^ A study in guinea pigs found that baseline choroidal parameters could predict future myopia development.^172^ Although numerous individual studies have reported a link between emmetropization and the choroid, there are still no widely accepted mechanisms to explain its role.

### The Sclera

Increased attention has been given to the sclera, as it is the final tissue in the cascade leading to axial elongation. In the past 2 years, novel scleral proteins and signaling factors have been identified in experimental myopia. Additionally, several studies have focused on inflammatory mediated processes.

In mice, it was shown that form-deprivation myopia promoted sclera M2-type macrophage polarization, and that panobinostat-mediated inhibition of M2 macrophage polarization decreased myopia progression. These findings suggest that M2 macrophages are crucial in controlling extracellular matrix remodeling by fibroblasts.^173^ Other studies using form-deprived mice have suggested that ADAMTS1 and ADAMTS5 proteins are involved in hypoxia-related scleral remodeling,^174^ thrombospondin-1 plays a role in maintaining the homeostasis of scleral extracellular matrix,^175^ and C5b-9, which regulates the NLRP3 inflammasome, is upregulated.^176^ In tree shrews, it was found that levels of tumor necrosis factor-α, interleukins-6 and 8, monocyte chemoattractant protein-1, and nuclear factor κB were increased in form deprived eyes, further demonstrating a role of inflammation in myopic axial elongation.^177^

Besides inflammatory pathways, other recent findings implicate specific scleral mechanisms in myopia. It was reported that, in guinea pigs, scleral endoplasmic reticulum stress, induced by tunicamycin, resulted in myopic changes, altered scleral collagen, and decreased expression of collagen I.^178^ In mice, exogenous all-trans retinoic acid was shown to induce myopia and alter scleral biomechanics.^179^ In guinea pigs, brimonidine, an intraocular pressure-lowering agent that influences scleral remodeling, was effective in reducing form deprivation myopia.^180^

### Other Molecular and Physiological Findings

Numerous studies have recently evaluated other molecular and physiological pathways in relation to myopia. To briefly summarize, studies in mice used RNA-sequencing to identify a role of mitochondrial energy metabolism and immune cell activation,^181^ epitranscriptomics demonstrated altered A-to-I RNA editing,^182^ and a role of miR-671-5p was identified.^183^ Connexin 36 was implicated in myopic mouse retina with respect to retinal ganglion cell encoding.^184^ Additional evidence for a role of dopamine was demonstrated; in mice, it was reported that retinal dopamine D1 receptors were essential for controlling ocular growth and myopia development.^185^

These recent advancements in our understanding of myopia gained from animal models have highlighted novel mechanisms involved in eye growth regulation. Innovations have included expanded experimental tools. Findings emphasize the role of light characteristics in eye growth, and novel opsins, like OPN3, OPN4, and OPN5, are increasingly implicated in non-image-forming pathways regulating myopia. With the identification of growth factors, proteins, and molecules in experimental myopia, the choroid and sclera show promise as therapeutic targets. These findings collectively illustrate the interplay among environmental cues, genetic pathways, and tissue-specific mechanisms in myopia development and provide a basis for future investigation.

## Onset and Progression of Myopia in Young Adults

Several important papers on adult myopia progression have been published since the IMI white paper review^186^ on the topic. As noted in that review, data on axial elongation in adults are scarce. Researchers in Western Australia have since used cross-sectional and longitudinal data to evaluate axial length in children and adults. They analyzed data from eight studies across different age groups in Australia, and of particular interest were the longitudinal data from the Raine study in which adult axial lengths were evaluated at the age of 20 years and then re-evaluated at the age of 28 years.^187^ At the 20-year examination, there were 178 subjects with myopia among the 727 participants. Considering only those of European descent, axial length increased by 0.03 mm per year in myopes compared with 0.01 mm per year in non-myopes. This translates to 0.3 mm per decade, which would be expected to produce an approximate refractive change of −0.75 D.

The issue of environmental factors was examined using data on 624 participants in the aforementioned Raine study, 144 of whom were myopic when they were 20 years of age.^188^ Baseline myopia was associated with a greater myopic shift over the subsequent 8 years. Likewise, longer baseline axial length was associated with greater axial elongation. Greater myopia shift was also associated with female sex, reduced sun exposure (measured objectively using conjunctival ultraviolet autofluorescence), increased screen time, and higher polygenic score.^189^ The same was true for higher axial elongation, but the number of myopic parents also played a role. The authors note that whereas computer screen time and sun exposure were significant modifiable risk factors, they only accounted for around 1% of the variance in myopic shift.

A number of clinic-based analyses have been published in the last year. Although axial length data were not available, and progressing patients seek more frequent examinations, they provide useful insights. A retrospective analysis of 354 patients with myopia from the Brien Holden Vision Institute clinic in Sydney, Australia,^190^ suggests that between the ages of 18 and 30 years, myopia progresses by almost a diopter. Progression was more common in those working or studying in an academic environment compared with those in non-academic settings.^190^

The Dublin, Ireland, myopia research team analyzed data from over one million practice visits from 40 optometry practices across Ireland, identifying myopia in 18,620 patients, aged between 18 and 39 years at their first recorded examination, and with at least 2 visits more than 11 months apart.^191^ The overall median annualized myopia progression was −0.05 D per year for existing patients with myopia, −0.06 D per year for patients with existing high myopia, and −0.11 D per year for patients with incident myopia. The authors stress that refractive error is stable in the vast majority of patients with myopia but a sizeable proportion of younger adults and those with high myopia of all ages do progress at a clinically meaningful rate. Furthermore, progression was nearly 3 times as common in those aged 18 to 24 years compared with those aged 40 to 44 years.

A much higher rate of progression was reported in India for a practice-based sample of 2683 patients aged 18 to 30 years.^192^ The annual progression was approximately −0.25 D per year, with higher progression associated with younger age and higher myopia. It should be noted that these were patients seeking a refractive surgery consultation, and the mean level of myopia was approximately −5 D compared with −2 D in the sample from Ireland.^191^

Finally, a team of researchers^193^ explored evidence for myopia progression between the ages of 20 and 50 years using 3 datasets—the US National Health and Nutrition Examination Survey (NHANES)^194^ and 2 from clinical populations—one in Germany^195^ and one in Japan.^196^ In all three cases, there was evidence for approximately –1 D of myopia progression between the ages of 20 and 50 years. The authors further revisited the NHANES data and concluded that 19% of the population exhibit adult-onset myopia.^197^

## Conclusions

The IMI 2025 Digest highlights significant advancements in our understanding of myopia, with important implications for both clinical practice and research. A central theme across the updated sections is the growing body of evidence supporting earlier identification and intervention for myopia, particularly in the pre-myopic phase.

The definitions and classification of myopia section reinforces the original IMI definition of pre-myopia while further exploring the nuances of each component of the definition. The expanding body of evidence underscores the importance of cycloplegic refractive error in identifying children at risk, with growing emphasis on the concept of hyperopic reserve as a predictive marker. Notably, the threshold for hyperopic reserve may vary across populations, particularly in Asia, pointing to the need for region-specific reference values. Importantly, we distinguish between risk factors and predictive factors for pre-myopia, noting that although many children exhibit risk factors, only some will progress, hence the critical role of accurate prediction. Encouragingly, recent studies suggest that interventions during the pre-myopic years can significantly reduce the incidence of myopia, presenting a compelling case for prevention-focused care.

Aligned with these findings, updates to clinical management guidelines stress that each year of delay of onset may be more impactful than several years of treatment. As predictive tools such as online calculators become more sophisticated and accessible, we are better positioned to identify children at genuine risk of myopia onset. However, this also brings a responsibility to balance the benefits of early intervention with the risk of overtreatment (that is, initiating myopia control in children unlikely to develop clinically significant myopia, potentially exposing them to unnecessary treatment burden, costs, or side effects). With more precise prediction, we can mitigate this concern and achieve improved population-level outcomes.

The updated section on risk factors for myopia explores why many current studies remain limited in their ability to infer causality. However, methodological advances, such as MR, RDA, and instrumental variable analysis, offer new pathways for disentangling causal relationships. Although the associations between myopia and behaviors such as sleep and screen time remain inconclusive, the COVID-19 pandemic has further reinforced the impact of environmental exposures, supporting earlier IMI conclusions that time spent outdoors remains among the most influential modifiable factors.

For accommodation and binocular vision, recent data confirm prior findings: there is no consistent association between these functions and myopia onset or progression. Moreover, evidence from clinical trials continues to show that myopia control treatments exert minimal impact on binocular and accommodative function, suggesting these factors should not be a barrier to intervention.

In the field of experimental models, recent work has revealed new light-regulated mechanisms underlying ocular growth, with non-image-forming pathways involving opsins, such as OPN3, OPN4, and OPN5, emerging as important players. Animal models also continue to uncover a growing list of molecular mediators, including growth factors and proteins, especially in the choroid and sclera, identifying these tissues as potential therapeutic targets. These advances collectively highlight the complex interplay among environmental cues, molecular signaling, and structural changes in the eye.

Finally, new data on young adult myopia, particularly from Australian cohorts, support previous findings that a proportion of younger adults and high myopes, especially those with longer axial lengths or higher baseline refractive error, continue to progress at clinically meaningful rates. These findings affirm the importance of ongoing monitoring, and potentially slowing progression, beyond childhood, especially for higher risk individuals.

In summary, the IMI 2025 IMI reflects a field that is rapidly advancing across multiple dimensions, from predictive precision and preventive care to mechanistic understanding and long-term management. These updates support a more proactive and individualized approach to myopia care and provide a strong foundation for future research, innovation, and global public health strategies.
